# Oral Care in Head and Neck Radiotherapy: Proposal for an Oral Hygiene Protocol

**DOI:** 10.3390/jpm14091013

**Published:** 2024-09-23

**Authors:** Giacomo Spinato, Valentina Schiavon, Sara Torvilli, Stefano Carraro, Federica Amato, Antonio Daloiso, Adolfo Di Fiore, Vittorio Favero, Leonardo Franz, Gino Marioni, Cosimo de Filippis, Cristoforo Fabbris, Enzo Emanuelli, Piero Nicolai

**Affiliations:** 1 Department of Neuroscience, Section of Otolaryngology, University of Padova, 35121 Padova, Italy; 2 Department of Neuroscience, School of Dentistry, University of Padova, 35121 Padova, Italy; valentina.schiavon.2@studenti.unipd.it (V.S.);; 3 Department of Neuroscience, Section of Maxillofacial Surgery, University of Padova, 35121 Padova, Italy; vittorio.favero@unipd.it; 4 Department of Neuroscience, Section of Audiology and Phoniatry, University of Padova, Treviso Hospital, 31100 Treviso, Italygino.marioni@unipd.it (G.M.); cosimo.defilippis@unipd.it (C.d.F.); 5 Department of Medicine DIMED, University of Padova, 35122 Padova, Italy; 6 ENT Unit, Department of Surgery, South Padova United Hospitals, 35043 Padova, Italy; 7 AULSS 2 Marca Trevigiana, Section of Otolaryngology, Treviso Hospital, 31100 Treviso, Italy

**Keywords:** radiotherapy, head and neck cancer, oral cavity, hygiene

## Abstract

This review aims to provide a comprehensive overview of the literature on the oral side effects caused by radiotherapy for head and neck cancers. Various treatments are examined to mitigate these sequelae, and a protocol is proposed for dentists and dental hygienists to manage oncological patients. A literature search was conducted to select relevant articles addressing the effects of radiotherapy treatments on the oral cavity, with a particular focus on the development of mucositis, candidiasis, changes in salivary pH, trismus, fibrosis, and alterations in the oral biofilm. PubMed and MedLine were used as search engines, with keyword combinations including: head and neck cancer, mucositis, candida, dental care, dental hygiene, epidemiology, oral microbiome, biofilm, trismus, fibrosis, and salivary pH. A total of 226 articles were identified, spanning the period from 1998 to 2023. Articles deemed inappropriate or in languages other than English or Italian were excluded. A management protocol for oncological patients was proposed, divided into two phases: home-based and professional. Despite the advancements in intensity-modulated radiation therapy, it is impossible to completely avoid damage to healthy tissues. Preventive education and counseling in the dental chair, ongoing motivation, and education about oral hygiene are crucial to combine a good therapeutic outcome with an improved quality of life for the patient.

## 1. Head and Neck Cancer Epidemiology

Neoplasms of the head and neck region represent the sixth most common group of cancers, with an estimated increase of over 30% by 2030 as reported in the literature [1,2]. Males are at a risk up to four times higher of developing the disease than females. The average age at diagnosis is estimated to be around 66 years, unless associated with viral infections. When considering the rising infections due to HPV, the age drops to 53 years for HPV-associated oropharyngeal cancers and to 50 for EBV-associated nasopharyngeal neoplasms [3,4,5,6,7,8]. Thanks to the development and refinement of diagnostic techniques, which allow for earlier stage detection, and advancements in treatment, including more precise and effective radiotherapy protocols, the five-year survival rate has significantly increased over the last 30 years, going from 55% in 1992–1996 to 66% in 2002–2006. This improvement is also attributed to a multidisciplinary approach in patient care, encompassing better supportive therapies and a deeper understanding of the disease’s biology [9,10]. However, despite treatments becoming increasingly targeted and less debilitating, these anatomical regions often suffer a significant negative impact on aesthetics and phonatory, respiratory, swallowing, and masticatory functions. This results in psychological and social discomfort, affecting the patient’s quality of life [11,12].

## 2. Purpose of the Study

The purpose of this review is to propose a protocol for both home and professional dental hygiene, using a comprehensive overview of literature on the side effects in the oral cavity caused by radiotherapy (RT) for head and neck cancers. Various treatments to ameliorate these sequelae are analyzed, and a protocol is proposed for dentists and dental hygienists, where they can play a significant role in managing oncological patients.

## 3. Materials and Methodology

A comprehensive literature review was conducted with the aim of selecting pertinent articles that discuss the effects of radiotherapy treatments on the oral cavity, with a particular focus on the development of mucositis, candidiasis, alterations in salivary pH, trismus, fibrosis, and changes in the oral biofilm. PubMed and MedLine were used as search mechanisms, using combinations of keywords that included: head and neck cancer, mucositis, candida, dental care, dental hygiene, epidemiology, oral microbiome, biofilm, trismus, fibrosis, management, treatment, and salivary pH.

Articles focusing on human subjects and the aforementioned aspects were included.

Articles that did not pertain to human subjects, were not in English or Italian, or did not address the research topic were excluded.

## 4. Results

A total of 226 articles were identified, spanning the period from 1998 to 2023.

After the removal of duplicates and the exclusion of 147 records, 79 articles relevant to the topic were examined. A total of 64 studies did not meet the inclusion criteria or were unavailable for retrieval. Finally, 15 were included in the review.

The side effects analyzed across various studies were categorized into acute-onset side effects and late onset side effects. Acute-onset side effects include mucositis, candidiasis (C.), xerostomia, dysgeusia, and dysphagia.

Late-onset side effects include osteoradionecrosis, caries occurrence, and fibrosis, which can affect the irradiated skin, muscles, and joints (Table 1).

**Figure 1:**
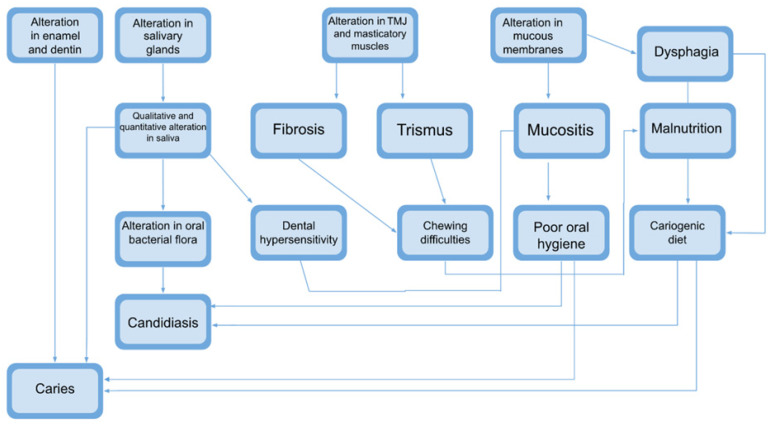
Evolution of damage caused by radiotherapy: This figure illustrates the interconnections among various complications.

All of these effects, as can be observed in Figure 1, are closely interrelated.

## 5. Discussion

Although RT is one of the most commonly used treatments for head and neck cancer, it is burdened by various adverse effects that may occur at varying rates during therapy [13]. Acute and late toxicities are summarized in Table 1. The oral microbiome, with over 700 species of bacteria, fungi, viruses, and protozoa, plays a crucial role in systemic health and potential development of inflammatory diseases [14]. The immune system tends to first modify the bacterial flora based on the gingival inflammatory response or the secretory antibody immunity in saliva. Numerous studies have underscored the pivotal role of the oral microbiome in the development and progression of head and neck cancers [15]. In particular, two species of Streptococcus (*mitis* and *thermophilous*) capable of degrading alcohol into acetaldehyde, a known carcinogen, are correlated with malignancy [16]. Beyond this mechanism, their ability to degrade mucin, and thereby further compromise the protective layer of the microbiome, plays a carcinogenic role [17]. Through similar mechanisms, poor hygienic conditions, and tobacco and alcohol consumption also contribute to increased bacterial adhesion.

### 5.1. Oral Mucositis

**Figure 2:**
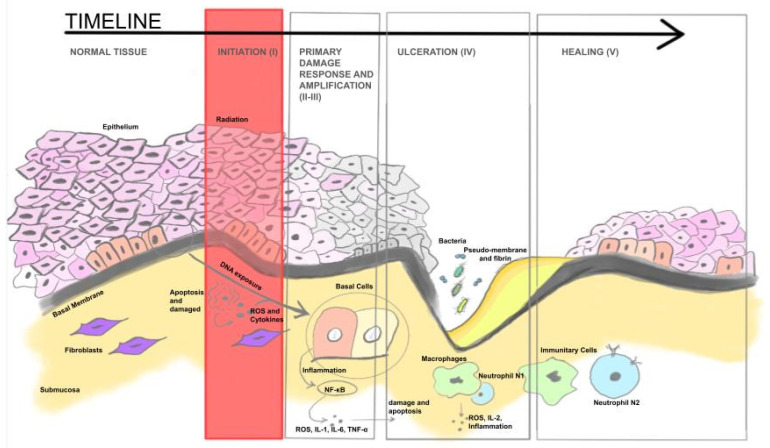
This figure elucidates the progression of tissue damage and the subsequent healing mechanism. The initial stage (I) involves tissue radiation, leading to the generation of ROS (reactive oxygen species) and cytokines. The second and third stages (II–III) encompass the activation of inflammation genes (such as NF-κB—“nuclear factor kappa-light-chain-enhancer of activated B cells”) and cells, resulting in the production of IL-1-6 (interleukins 1-6) and TNF-α (tumor necrosis factor α). The fourth stage (IV) is characterized by tissue colonization and the formation of pseudomembrane and fibrin (N1—pro-inflammatory neutrophils). The final stage (V) culminates in the restoration of healthy tissue (N2—anti-inflammatory neutrophils).

One of the earliest and most apparent side effects of RT in the head and neck region is inflammation and subsequent deterioration of the oral mucosa. The initial signs of oral mucositis (OM) typically manifest after cumulative doses between 15 and 20 Gy [18], reaching peak severity around 30 Gy. The extent of mucosal damage varies depending on the dose of radiotherapy and its fractionation, ranging from erythema to actual ulceration. Mucosal inflammation generally peaks around 2 to 4 weeks following the commencement of radiotherapy, with recovery lasting several weeks, particularly if RT is combined with chemotherapy or systemic therapy. The lesions cause intense pain, to the extent of impairing phonation and swallowing, necessitating the use of a nasogastric tube for adequate patient nutrition and prevention of significant weight loss. Ulceration, coupled with reduced saliva production and loss of its buffering capacity (an effect of RT on the salivary glands), also facilitates enhanced adherence and, thus, increased colonization by pathogens, primarily enteric bacteria (already present in the oral cavity as commensals), Gram-negative anaerobes, and yeasts [19] (Figure 2).

The most severe form of mucositis is associated with colonization by enteric bacteria from the Enterobacteriaceae family, which possess proteolytic activities that further exacerbate the clinical condition.

The reduction of blood flow to irradiated areas due to vascular fibrosis also results in a diminished immune response and impaired tissue healing, creating an optimal environment for the proliferation of obligate anaerobic bacteria, such as *Fusobacterium*, *Porphyromonas*, *Prevotella*, and *Treponema* [20]. A study conducted by Cielito et al. [21] investigated the bacterial populations associated with varying degrees of mucositis, finding that the presence of certain bacterial species (such as *Cardiobacterium* and *Granulicatella*) and a lower quantity of *Streptococcus* is linked to the early onset of severe mucositis [22]. Radiation treatment alters the species of *Streptococcus* (S.) present in the oral cavity, such as *S. salivarius* and *S. mitis* [23], and with the increase of *Fusobacterium* and *Prevotella*, it is associated with altered oral pH and an increased risk of severe mucositis. Currently, there are no definitive therapies for treating radiation-induced mucositis, due to the biological complexity attributed to its pathogenesis.

There are numerous dynamic mechanisms at the onset of radiation-induced damage. Specifically, the damage mechanism differs between healthy and diseased cells, leading to genomic and macrobiotic alterations [24,25,26,27]. The type of irradiated cells also plays a significant role; for instance, keratinized cells endure less damage than mucous membranes [28]. Previous chemotherapy or radiotherapy also exerts a detrimental effect.

Based on the reviewed literature, current treatment options for OM involve a combination of patient education, medical therapy, and surgical therapy. Patient education includes proper oral hygiene practices such as using soft-bristled toothbrushes and rinsing with alcohol-free mouthwashes or sodium bicarbonate-based solutions [29]. Therapies for radiation-induced mucositis include the use of anti-inflammatory agents such as benzydamine [30], celecoxib, irsoglandine maleate, misoprostol, and rebamipide. However, these agents have not shown statistically significant influence in studies to be included in this proposed management protocol [31]. Antimicrobials such as fluconazole, miconazole, and sucralfate reduce the risk of tissue infiltration by pathogens present in the oral microflora. Analgesics, including the topical application of morphine and fentanyl, may provide symptomatic relief. However, it is crucial to be aware of the potential for addiction and dependence associated with these medications, especially when used chronically. Coating agents such as mucoadhesive hydrogel and sucralfate [32] are used to alleviate pain associated with radiation-induced mucositis. Doxepin is used to reduce itching sensations [33,34]. Furthermore, photobiomodulation (PBM, or photobiomodulation therapy, PBMT) with LED lasers promotes tissue regeneration by increasing blood supply to the tissues, reducing the severity of mucositis, and delaying its onset through improved inflammatory exudate drainage and inhibition of pro-inflammatory cytokine production. It also allows better pain control through the depolarization of membrane receptors that conduct pain stimuli [35,36]. Another strategy to manage the oxidative stress of mucositis is the use of epigallocatechin-3-gallate, a molecule present in Kariosyte mouthwash.

Among surgical treatments, cryotherapy exploits the vasoconstriction of the superficial microcirculation in oral tissue to limit the absorption of cytotoxic drugs used, potentially minimizing oral mucosa inflammation by slowing down epithelial metabolism. This procedure is performed by maintaining ice/cold water within the oral cavity during the administration of chemotherapy doses [37].

Various studies suggest that natural substances appear to promote the healing of ulcerations. These include dietary supplements containing zinc, vitamin E, selenium, calcitriol, and glutamine. Additionally, oral rinses with calcium phosphate, chamomile, ford, calendula, olive leaf extract, mallow, and moose have also been proposed [38,39,40,41,42,43].

On the other hand, some authors have conducted studies on the beneficial effects of decoctions containing Qingre Liyan, rhubarb, licorice, mint, and Japanese lily-of-the-valley root in the treatment of mucositis, following a suggestion from Chinese medicine [44,45]. However, there has not been sufficient evidence to include these types of treatments in the proposed treatment protocol [46,47].

### 5.2. Oral Candidiasis

Oral and pharyngeal candidiasis is one of the most common side effects, as alterations in the oral microbiome induced by radiotherapy treatment foster the colonization of pathogenic microorganisms [48,49,50].

Virulence factors of this mycete include the following:

- The expression of cell wall proteins (such as adhesins and secreted aspartate-proteinases) facilitates adhesion to epithelial cells and the extracellular matrix. This process is further enhanced by the hydrophobicity of the cell membrane, which enables the generation of exceptionally strong electrostatic forces [51];
- The expression of surface antigens similar to molecules of the human complement system allows the microorganism to camouflage itself and evade the host’s immune response, which is already weakened by the lysis of cytokines involved in the host’s cellular defense by aspartate proteinases secreted [52];
- The ability to transition from the blastoconidia phenotype to hyphae, which enhances its tissue invasion capacity and hinders its phagocytosis [53];
- Biofilm formation not only enhances surface adhesion but also provides protection and promotes the development of more virulent complex fungal communities, such as *C. tropicalis* and *C. glabrata* [54].

Clinically, the onset of oral candidiasis is associated with oral cavity pain, dysgeusia, and when extended to the esophagus, dysphagia. Macroscopically, two primary forms can be distinguished: chronic erythematous candidiasis, characterized by erythematous lesions typically at the level of the palate, and chronic hyperplastic candidiasis, which presents as a thickened white plaque at the level of the labial commissures or on the dorsum of the tongue. Treatment options proposed in the literature involve a combination of oral hygiene and pharmacological therapies. Adequate oral hygiene is essential for biofilm removal, achieved through the mechanical action of tooth brushing combined with the use of mouthwashes containing chlorhexidine or triclosan [55]. It is also crucial to clean removable prosthetic restorations with peroxide, hypochlorite, or ethylenediaminetetraacetic acid (EDTA). Pharmacological therapy for oral candidiasis employs topical antifungals such as nystatin, amphotericin, miconazole, and clotrimazole, and systemic antifungals such as ketoconazole, fluconazole, and itraconazole [55,56].

Salivary glands are highly susceptible to radiation-induced damage and are often included in the irradiation field. The primary manifestation of such damage is the alteration in saliva production, both in terms of quantity and quality. Not only is saliva produced in lesser amounts, but it also becomes more viscous due to the altered structure of mucin, which reduces its ability to bind water molecules. Additionally, it becomes more acidic, affecting its buffering properties with an altered electrolyte concentration [57,58]. Saliva electrolyte alterations encompass various changes; the reduction in potassium concentrations and the increase in chlorine result in a decrease in neurotransmitter release by neurosensory cells that recognize bitter and sour tastes, leading to dysgeusia. Conversely, the decrease in calcium impedes the adequate remineralization of dental tissues, resulting in an increase in carious lesions [59].

### 5.3. Xerostomia

The primary symptom associated with glandular tissue damage is xerostomia, which impairs mastication and swallowing, and diminishes the patient’s quality of life in relational terms [60]. Numerous studies investigating potential treatments for salivary alterations have demonstrated benefits from the use of medications such as pilocarpine in the form of chewable tablets or oral rinse solutions with a 0.1% concentration [61]. Furthermore, the use of hyaluronic acid allows for the formation of cross-linked mucin, promoting water absorption and thus hydration. Alternatively, the use of artificial saliva, such as water-based products fortified with electrolytes and humectants, can be considered.

### 5.4. Caries

Another consequence of head and neck irradiation is the increased incidence of caries lesions. The direct effect of radiation on the tooth contributes to the genesis of this complication, with the degeneration of odontoblastic processes creating a gap at the enamel-dentin level [62], and an indirect effect related to changes in salivary composition. Saliva, as noted, causes a decrease in pH, a reduction in calcium ion concentrations, and an increase in cariogenic bacterial species, leading to enamel demineralization. Clinical evidence supported by various studies has shown that irradiation of hard tissues results in a higher likelihood of enamel loss and a greater occurrence of carious lesions. Beyond direct damage, the patient’s psychological state also impacts the maintenance of proper oral hygiene, further exacerbating the problem. Although data on the incidence of radiation-dependent carious disease are heterogeneous due to the variables considered by different studies, results obtained by Moore and colleagues estimated that carious lesions occur in approximately 29% of post-radiotherapy head and neck cancer patients, and the risk of developing caries within two years post-radiotherapy is about 37% [63]. Treatment options for caries in patients undergoing radiotherapy include oral hygiene to decrease the bacterial load responsible for cariogenesis, occupational fluoroprophylaxis, and home fluoroprophylaxis through the application of fluoride and/or hydroxyapatite-containing gels/trays/toothpastes. Limiting the consumption of cariogenic foods (sweets, acids, and spices) is also beneficial.

### 5.5. Osteoradionecrosis

Late complications of radiotherapy, on the other hand, encompass osteoradionecrosis and tissue fibrosis. The etiopathogenic basis of osteoradionecrosis appears to be the direct radiation damage to the bone, which triggers a tissue remodeling process. However, this process is not adequately sustained by the blood supply, which is reduced due to radio-induced vascular fibrosis. This damage results in a state of chronic inflammation with the formation of fibrous tissue that is more fragile than bone [64]. This could explain why the mandible, which has a larger blood supply than the maxilla under physiological conditions, is more affected [65]. Other factors such as the dose and fractionation of radiotherapy, dental extractions, poor oral hygiene, age, smoking, periodontitis, diabetes mellitus, and reconstructive bone flaps also contribute to its genesis. Clinical manifestations include pain, swelling, and, in severe cases, ulceration of soft tissues with exposure of the necrotic bone portion and the occurrence of fractures. It generally develops between 18 and 24 months after radiation treatment, for cumulative doses exceeding 54 Gy according to some authors, and over 60 Gy according to others [66,67,68]. Regarding the treatment of osteoradionecrosis, a combination of oral hygiene, drug therapy, oxygen therapy, and surgical treatments is reported in the literature [69]. Oral hygiene is crucial to avoid the extraction of compromised dental elements, which could increase the risk of fracture, and to prevent excessive infection of the necrotic tissue [70,71]. Drug therapy is combined with the use of clodronate, which inhibits bone resorption by osteoclasts, pentoxifylline, which improves microcirculation and tissue oxygenation, and tocopherol (vitamin E), which reduces the formation of oxygen free radicals and inhibits the production of pro-inflammatory cytokines such as TNF-α (tumor necrosis factor α) and TGF-β (tumor growth factor β) [72,73,74]. Surgery is typically combined with the exposure of necrotic bone portions and the removal of non-viable bone parts reconstructed with mucosal flaps [75]. Hyperbaric oxygen therapy benefits from its ability to stimulate neoangiogenesis and consequently osteogenesis, in addition to its bacteriostatic-bactericidal and anti-edematous action. Hyperbaric oxygen therapy is often associated with surgery, with which it appears to have a synergistic effect.

### 5.6. Fibrosis

Finally, a long-term side effect of radiotherapy that warrants mention is tissue fibrosis. The pathogenesis of this condition is linked to the establishment of a chronic inflammatory state that stimulates the migration of fibroblasts to the irradiated tissue, leading to an overproduction of fibrous tissue [76,77,78,79,80]. The incidence rate in patients treated with IMRT (intensity-modulated radiation therapy) is 30%, which increases to 34% when combined with chemotherapy and to 50% when combined with surgery. Depending on the location, it can lead to various outcomes, such as difficulty in chewing and swallowing if it affects the tongue muscles and pharyngeal constrictors, or cause trismus if it affects the masticatory muscles and the temporomandibular joint [81,82,83]. Trismus and dysphagia are associated with a decline in the patient’s quality of life due to malnutrition and reduced oral hygiene. They are also linked to stiffness and postural problems due to fibrosis in the neck and shoulder muscles [84,85].

Fibrosis of the irradiated district, which commences with erythema a few weeks post-radiation treatment, followed by depigmentation, atrophy, and fibrosis, can, in severe cases, result in tissue ulceration [85].

Radiotherapy-induced scar fibrosis-related symptoms can be reduced by:

- Laser photobiomodulation [86];
- Jaw mobilization devices, which are beneficial for expanding the interincisal opening and reducing complications associated with trismus [87];
- Pharmacological means, through the use of pentoxifylline and tocopherol, which, due to their anti-inflammatory and vasodilatory actions, are capable of enhancing the blood and oxygen supply to tissues, thereby reducing fibrogenesis. Additionally, botulinum toxin injections can prevent spasms of the masticatory muscles at the base of trismus [88].

### 5.7. Protocol for Dental Management of a Head and Neck Cancer Patient

Following an analysis of the therapeutic options used to mitigate the side effects of radiotherapy on the oral mucosa, dental tissues, musculoskeletal structures of the head and neck, and oral microflora, we propose a protocol for the dental management of cancer patients. The damage to healthy tissues cannot be entirely prevented, although modern imaging techniques and radiotherapy allow for improved management of radiation in terms of quantity and spatial distribution. It is crucial to understand the potential complications associated with radiation and/or chemotherapy treatment in order to delay their onset, alleviate their symptoms, and avoid systemic drug treatment in frail individuals [89]. The following treatment proposal serves a preventive/palliative purpose and should be perceived as a general therapeutic guideline to be tailored to each patient’s specific needs and individual capacities. The protocol is divided into two distinct phases: a home-based phase and a professional phase.

### 5.8. Home Oral Hygiene and Recommended Devices

The choice of home oral hygiene devices should focus on the ease of plaque removal and control by causing as little trauma as possible to already compromised tissues.

Manual toothbrushes should possess the following characteristics: small heads with beveled edges to minimize tissue trauma; a comfortable and ergonomic handle; and curved bristles selected according to the degree of gingival mucosa injury and patient comfort (soft, super soft, ultra soft).

The use of an electric toothbrush may be considered if there are no lesions present, or if any lesions are in an early stage. The toothbrush should be equipped with a pressure detection system, and the brush heads should be round, featuring a rotating–oscillating movement. The bristles should be extra soft and should not include silicone inserts.

The dental floss recommended should be fluffy to optimize the removal of bacterial plaque and food residues without traumatizing the gingival mucosa. The use of waxed, tape, or expandable dental floss should be avoided.

In the case of patients with periodontal disease or large interdental spaces, it is particularly recommended to use pipe cleaners equipped with Tynex bristles and a spiral metallic core. The size of the pipe cleaners should be carefully selected according to the individual characteristics of each patient.

To aid in reducing the overall bacterial load, it is advisable to clean the tongue following routine domestic oral hygiene practices using a bristle-equipped tongue cleaner. Its use should be limited to the absence of lesions on the tongue’s dorsum and/or any discomfort expressed by the patient (Table 2).

The prescribed toothpaste should possess a low abrasiveness index and contain fluoride and/or hydroxyapatite (fluoro-hydroxyapatite, Mg-Sr carbonate, zinc-substituted carbonate-apatite, chitosan-conjugated hydroxyapatite) to promote enamel mineralization and reduce dentin sensitivity resulting from erosion. Formulations containing 0.12% chlorhexidine and 0.05% CPC are available to assist in biofilm disruption. It is advisable to select a toothpaste devoid of sodium lauryl sulfate, as it can be irritating to mucous membranes. The toothpaste should be applied by gently cleaning the surfaces after each meal.

The use of alcohol-free mouthwashes containing chlorhexidine (0.12% for extended treatment or 0.20% for intensive treatment) is recommended, potentially enriched with CPC (0.05%). In instances of pain, a mouthwash fortified with chlorobutanol (0.5%) can be used to alleviate symptoms. In the presence of ulcerative lesions, mouthwashes with colostrum, PVP-VA, or hyaluronic acid will aid in healing. Market-available mouthwashes containing benzidine hydrochloride (0.15%) are beneficial for relieving inflammatory and irritative states of the mucosa. The application of the mouthwash should be performed by rinsing for 1 min, three times a day. There are also certain molecules that can be associated with the described treatments to protect the epithelium, such as epigallocatechin-3-gallate (Kariosyte twice daily). This molecule functions as an antioxidant and epithelial anti-apoptotic, particularly at the level of glandular ducts following radiation injury [90].

It is recommended to apply fluoride mousse to the tooth surfaces twice daily using a soft toothbrush. Wait for two minutes before expelling the excess product and refrain from eating or drinking for 30 min following the application.

In cases of xerostomia, the additional use of water-based substitutes in gel or spray formulations with fortified viscoelastic properties containing hyaluronic acid is anticipated. The application should be at least 3 to 4 times daily, as needed, with particular attention to ensuring good lubrication before bedtime. In instances of candidiasis, a suspension based on nystatin 100,000 I.U. can be used as a rinse 3 to 4 times daily, ensuring it is not combined with chlorhexidine as it tends to negate its effects. Nystatin is recommended for chronic erythematous candidiasis. An oral suspension of fluconazole 50 mg/5 mL, twice daily, can be used for candidiasis. The hygiene of removable prosthetic artifacts is important and cleaning should be performed after each meal using peroxide, hypochlorite, or tetrasodium (EDTA).

### 5.9. Professional Oral Hygiene Sessions

An initial professional oral hygiene session should be conducted 15 days prior to the commencement of treatment (Table 3). This session should encompass home oral hygiene instructions, nutritional counseling, bacterial plaque debridement following the use of a plaque detector to motivate the patient, removal of supragingival and subgingival tartar, comprehensive polishing of dental surfaces to minimize biofilm adhesion, measurement of maximum mouth opening, and fluoroprophylaxis.

A follow-up should be conducted one week after the initiation of treatment to monitor the control of bacterial plaque at home and to motivate the patient. Fluoroprophylaxis will only be performed if the patient does not use fluoride mousse at home.

Subsequently, oral hygiene sessions will be evaluated weekly until the end of radiotherapy. This is to assess the state of oral health, motivate the patient, measure the maximum mouth opening, and modify the treatment plan if necessary.

Fluoride prophylaxis will only be performed if the patient is not using fluoride mousse at home. Follow-up appointments will be scheduled 1, 2, and 3 months after the completion of the treatment. Procedures such as bacterial plaque debridement, tartar ablation, motivation for home oral hygiene, fluoride prophylaxis, and measurement of maximum mouth opening will be conducted. These are aimed at intercepting the early onset of trismus and referring the patient to the appropriate professional in a timely manner.

Recalls for professional oral hygiene sessions should be conducted every 3–4 months, monitoring the condition of the oral cavity.

Even with the most advanced IMRT methodologies, damage to healthy tissues cannot be completely avoided. However, this protocol should be integrated through a comprehensive patient evaluation within a multidisciplinary environment, involving the surgeon, oncologist, radiation therapist, nutritionist, infectious disease specialist, and pain management physician.

Prevention, education, dental chair counseling, consistent motivation, and oral hygiene education are fundamental to associating a positive therapeutic outcome with an enhanced quality of life for the patient.

### 5.10. Strength and Limitations of the Present Study

This study was conducted by the same team of experts. The information was obtained through the analysis of articles published in international literature.

Given the comprehensive nature of this review, standard review protocols were not adhered to, thus precluding the possibility of drawing a rigorous conclusion.

Early identification and intervention are vital in managing these complications effectively. Dental practitioners should be vigilant in monitoring patients for symptoms such as persistent pain, exposed bone, or limited jaw movement. Upon observing these signs, a prompt referral to an oral and maxillofacial surgeon or a head and neck specialist is recommended. These specialists can provide advanced diagnostic evaluation and implement targeted management strategies, potentially mitigating the progression of these serious conditions.

## 6. Conclusions

Despite the advancements in IMRT, radiation therapy is not devoid of potential harm to healthy tissues. To enhance the quality of life for patients, appropriate oral hygiene recommendations are imperative. The present study provides general guidelines to be followed to support oncology patients.

Even though dentists typically do not participate in a multidisciplinary tumor board, it is imperative that a comprehensive evaluation is conducted to assess and potentially treat patients. This may include procedures such as tooth extraction, imaging examinations, dental root treatment, and prosthesis management, among others.

Further research is required to propose and validate a protocol. Generally, counseling in the dental chair, preventive education, and consistent motivation are fundamental.

## Figures and Tables

**Table 1:** Acute-onset and late-onset complications of RT.

Acute Complications	Late Complications
Oral mucositis	Osteoradionecrosis
Disruption of the oral microbiome	Caries
Candidiasis	Skin fibrosis
Xerostomia	Muscles fibrosis
Dysphagia	Joint fibrosis
Dysgeusia	

**Table 2:** Treatment undertaken to prevent various complications from radiotherapy, along with the initiation time for each treatment.

	Toothpaste	Mouthwash	Drugs	Other
**From 15 days before** **of the start of** **treatment** **radiotherapy**	Fluoridated, SLS-free	Fluorinated, fortified with CPC and chlorhexidine		Mousse with fluoride (2 times/day)
**Onset of** **mucositis**	Fluorinated, SLS-free, fortified with chlorhexidine and CPC	Added with Benzidamine (1–2 times/day) Karyosite 2 times/day		
**Onset of** **candidiasis**	Fluorinated, SLS-free, fortified with chlorhexidine and CPC	Fluorinated, with chlorhexidine (1 time/day, away from Fluconazole)	Fluconazole suspension 50 mg/5 mL (2 times/day)	
**Onset of** **candidiasis (particularly** **particularly if** **HCC)**	Fluorinated, SLS-free, NOT fortified with chlorhexidine	Fluorinated, NOT fortified with chlorhexidine	Fluorinated, NOT fortified with chlorhexidine Nystatin suspension 100,000 I.U.	
**Onset of** **xerostomia**	Fluorinated, SLS-free, fortified with chlorhexidine and CPC	Fluorinated, fortified with chlorhexidine (3 times/day)		Substitute for saliva gel or spray with hyaluronan (3–4 times/day)
**After the end of the** **treatment** **radiotherapy**	Fluorinated, SLS-free	Fluorinated, fortified with CPC Karyosite 2 times/day		Mousse with fluoride (2 times/week)

**Table 3:** This timeline delineates the types of treatments that can be used to prevent complications.

	Ablation Tartar	Fluoroprophylaxis	Measurement Aperture Maximum Mouth	Rationale and Instructions IOD
15 days before of the start of the treatment	YES	YES	YES	YES
7 days before of the start of the tratment	As needed	If patient has not used fluorinated mousse	NO	As needed
1 time per week until the end of treatment RT	As needed	If patient has not used fluorinated mousse	YES	As needed
1 months from the end of the treatment RT (1st follow-up)	YES	YES	YES	As needed
2 months from the end of the treatment RT (2nd follow-up)	YES	YES	YES	As needed
3 months from the end of the treatment RT (3rd follow-up)	YES	YES	YES	As needed
Recalls of hygiene professional every 3–4 months	YES	NO	YES	As needed

Abbreviations: IOD = Induced oral dysfunctions; RT = radiotherapy.

## Data Availability

No new data were generated for this study.
